# Effect of aerobic and resistance exercise training on endothelial function in individuals with overweight and obesity: a systematic review with meta-analysis of randomized clinical trials

**DOI:** 10.1038/s41598-023-38603-x

**Published:** 2023-07-21

**Authors:** Maiquel Bueno Cortes, Raphael Silveira Nunes da Silva, Patrícia Caetano de Oliveira, Diego Silveira da Silva, Maria Claudia Costa Irigoyen, Gustavo Waclawovsky, Maximiliano Isoppo Schaun

**Affiliations:** 1 Instituto de Cardiologia do Rio Grande do Sul/Fundação Universitária de Cardiologia, Porto Alegre, Brasil; 2 Experimental Laboratory of Hypertension, Instituto do Coração (InCor), University of São Paulo (USP), São Paulo, Brazil; 3 Universidade Luterana do Brasil, Rio Grande do Sul, Brazil

**Keywords:** Physiology, Disease prevention, Quality of life, Weight management

## Abstract

The objective of this systematic review was to examine the effects of exercise training on endothelial function in individuals with overweight and obesity. Our review study included only randomized controlled trials (RCTs) involving adults (≥ 18 years of age) with body mass index (BMI) ≥ 25.0 kg/m_2_. Our search was conducted in the electronic bases MEDLINE (PubMed), Cochrane, LILACS and EMBASE and in the gray literature. We performed random-effects analyses for effect estimates and used 95% prediction intervals (95% PI) for estimating the uncertainty of the study results. There were selected 10 RCTs involving 14 groups (n = 400). The quality assessment of studies using Cochrane risk-of-bias 2 (RoB 2) tool identified some concerns. Exercise training resulted in improved flow-mediated dilation (FMD) in individuals with overweight and obesity (p < 0.001) compared to the no-exercise control group. This effect of training modalities on FMD was seen for aerobic training (p < 0.001) but not for resistance training (p = 0.051). There was no difference in FMD in response to exercise training by BMI classification (overweight, obesity, overweight + obesity), p = 0.793. The present results are consistent with the notion that aerobic exercise training elicits favorable adaptations in endothelial function in individuals with overweight and obesity. Our findings should be interpreted with caution because of the small number of studies included in this review.

## Introduction

Epidemiological data show an increase in the prevalence of overweight and obesity (BMI ≥ 25.0 kg/m^2^) worldwide^1^. It is concerning since overweight and obesity accounts for nearly 4.0 million deaths every year in developed countries and over half of them are due to cardiovascular events^2^. The risk of cardiovascular events is even higher when it is associated with comorbidities^3^, including metabolic syndrome, arterial hypertension and type 2 diabetes^4,5^.

The relationship between overweight and obesity with endothelial dysfunction and increased risk for cardiovascular events is already well established in the literature^6,7^. According to the last statement of the World Health Organization^8^, the exercise training recommendations for prevention of all-cause mortality, cardiovascular disease mortality, incident hypertension, among others metabolic disorders, are at least 150–300 min of moderate-intensity aerobic physical activity, or at least 75–150 min of vigorous-intensity aerobic exercise, throughout the week. The American College of Sports Medicine usually recommends for weight loss and prevention of weight regain for adults through moderate-intensity exercise, 30–60 min/session on 3–5 days a week, or vigorous exercise for 20–40 min on 3 days or more a week^9^. In this context, it is well recognized that one of the main cardiovascular benefits promoted by the practice of exercise training is the stimulus of the endothelial function for nitric oxide production^10^.

Endothelial nitric oxide (NO) plays a crucial role in vascular protection. It potentially reduces blood clot formation, exerts an anti-inflammatory effect, inhibits the proliferation of vascular smooth muscle cells and is essential for vascular tone control^11^. An increase in arterial diameter in response to vasodilating agents such as acetylcholine or mechanical stimuli such as shear stress is associated with endothelial NO production. Brachial artery flow-mediated dilation (FMD) is the gold standard, non-invasive approach to assess endothelial function^3,12^.

Overweight and obesity is a multifactorial condition, and its management involves changing eating habits and lifestyle, counseling^13^ and exercising^14^. A meta-analysis including 33 studies to examine the effect of weight loss from dietary changes alone, dietary changes with exercise training and drug intervention on FMD in individuals with overweight and obesity found that, irrespective of the intervention, each 10-kg decrease in body weight was correlated with 1.1% increase in FMD^15^. Interestingly, this effect is enhanced when associated with dietary changes, exercise training and drug therapy^15^. These findings are clinically relevant considering that 1% increase in FMD is associated with an 8–13% reduction in the risk of cardiovascular events^16^. Yet, the effect of exercise training alone was not examined and therefore we cannot draw any further conclusions about the benefits of this intervention in this population.

There is evidence pointing to improvement in FMD in response to aerobic^17^ and resistance training^10^ in individuals with cardiovascular risk factors. This training-induced benefit is largely due to increased shear stress in endothelial cells during exercise which promotes vascular adaptations and increased vasodilation and consequently improves endothelial integrity with greater bioavailability of NO^18^.

The findings of randomized clinical trials (RCTs) examining the effect of training on endothelial function in individuals with overweight and obesity are inconsistent. Some studies reported improvement in FMD^19,20^, others did not^21,22^. So far it is not known whether exercise training effectively improves endothelial function in individuals with overweight and obesity. Considering that an increase in FMD is associated with a reduction in cardiovascular risk^16^ and 12% risk reduction in future cardiovascular events^23^, it is clinically relevant to examine the effect of exercise training on FMD in this population.

Bearing in mind the heterogeneity of study findings and that we were not aware of other systematic review that have examined exercise training data in FMD among adults with overweight and obesity, we adopted a systematic review approach in the present study to provide a comprehensive overview of the effectiveness of exercise training interventions on FMD in this population. Thus, the primary objective of this systematic review was to examine the effectiveness of interventions involving aerobic or resistance training on FMD in adults with overweight and obesity.

## Methods

### Protocol and registration

**Figure 1:**
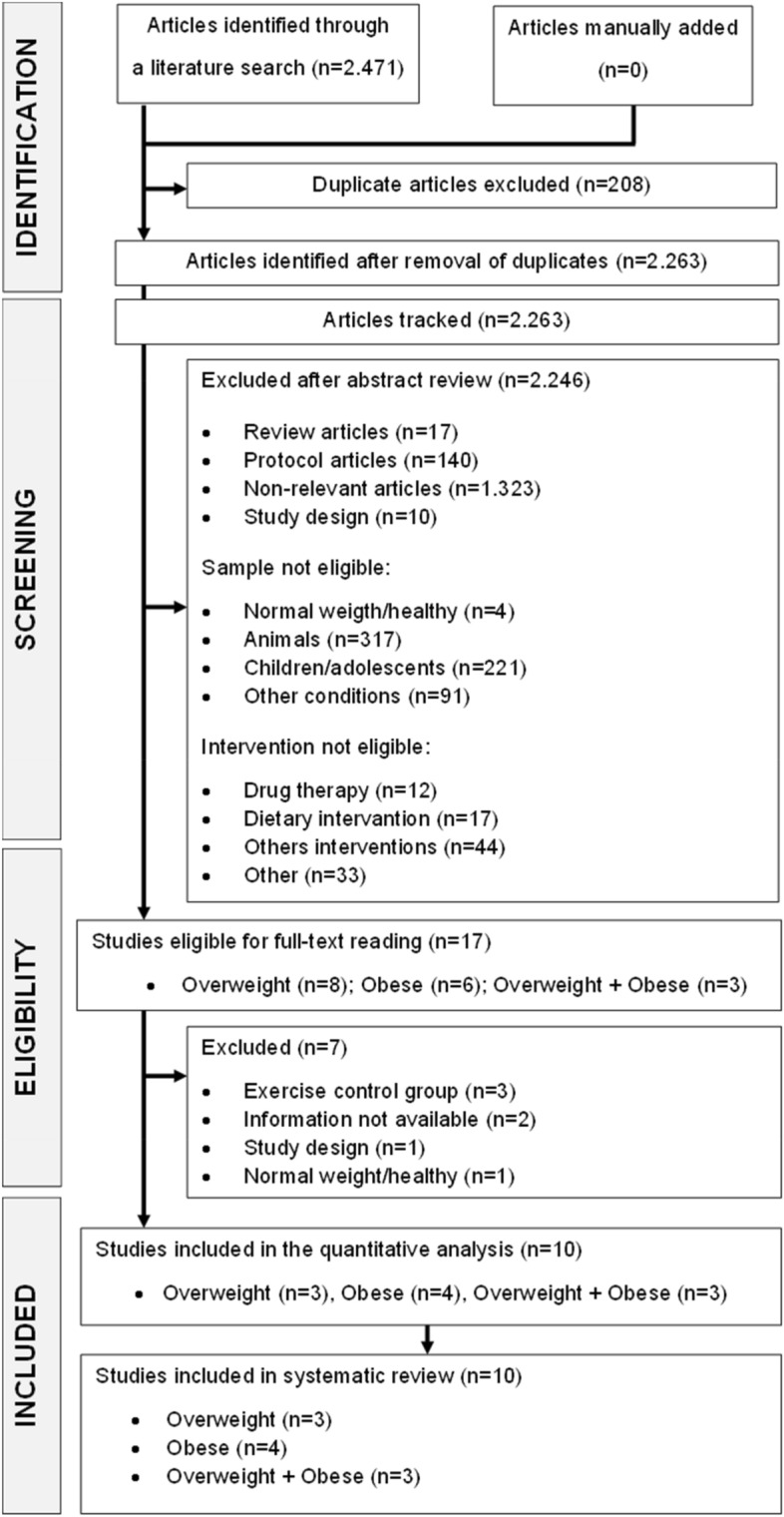
Flow chart summarizing the study selection process.

This systematic review with meta-analysis followed the recommendations PRISMA and Cochrane Handbook Higgins^24,25^ and was registered in the International Prospective Register of Systematic Reviews (PROSPERO) (CRD42020203397; registered on October 10, 2021; https://www.crd.york.ac.uk/PROSPERO/). Figure 1 is a flow chart that summarizes the study design. The database used for this meta-analysis is available on Mendeley Data repository (https://data.mendeley.com/) as open access under DOI (10.17632/94d9g5hx3v.3).

### Study design and eligibility criteria

We selected RCTs including both female and male adults (≥ 18 years of age) with overweight and obesity that examined the effect of exercise training interventions on FMD outcome published until July 2021.

All eligible RCTs were reviewed and selected by two independent reviewers (M.B. and R.S.N.S.) using the PICOS framework: Population (≥ 18 years of age with BMI ≥ 25.0 kg/m^2^); Intervention (aerobic or resistance exercise training); *Comparison* (exercise training vs. no-exercise control group); *Outcome* (endothelial function as measured by brachial artery FMD); and *Study* (RCT).

### Sources of information and search

First, two independent reviewers (M.B.C and R.S.N.S) searched the electronic databases MEDLINE (PubMed), Cochrane, LILACS, and EMBASE and screened the title and abstract for each of the study retrieved using the eligibility criteria. Our main MeSH terms were *overweight*, *obesity*, *exercise* and *endothelium*. Articles in any language with no date of publication limits were all eligible. For more accurate and sensitive results the search terms for RCTs were entered into the MEDLINE^26^ and EMBASE^27^ databases (Supplementary Table 1). To further our search, we searched online gray literature including OpenGrey and the Brazilian Coordination for the Improvement of Higher Education Personnel (CAPES) and Bank of Theses and Dissertations and the Brazilian Digital Library of Theses and Dissertations (BDTD). Another reviewer (D.S.S.) conducted searches for unpublished ongoing trials in the Brazilian Clinical Trials Registry (ReBEC), Clinical Trial.gov and WHO International Clinical Trials Registry Platform (ICTRP). Details on gray and unpublished literature searches can be found in the supplementary material (Supplementary Table 1). Any discrepancies were resolved through a consensus-based discussion and any disagreements were resolved by a third and a fourth reviewer (G.W. and M.I.S.).

### Study selection

To characterize individuals with overweight and obesity, only articles with a clear description of the study population as overweight and/or obese adults (BMI ≥ 25.0 kg/m^2^) were included. When information on the study population or results was not available the authors were contacted by e-mail and asked to provide additional information or clarification of data before any study was excluded in the selection process. The eligibility criteria for intervention studies were those with two or more arms involving aerobic or resistance exercise training compared with the no-exercise control group participants (primary care, usual care or wait-list control). There wasn't any restriction of language or data. Table 1 summarizes the main FITT (frequency, intensity, time and type) of exercise training interventions.

**Table 1:** Characteristics of the studies selected.

Study	Sample size (n)	BMI (kg/m^2^)	FMD baseline (%)	Exercise training	Main outcome	Secondary outcomes
Azadpour et al. (2016)^40^	Total = 24 Women = 24 T (n = 12): 58 years C (n = 12): 56 years	T: 32.2 ± 1.6 C: 31.3 ± 1.4	T: 5.8 ± 0.5 C: 6.0 ± 0.7	Aerobic, 25 to 40 min, 3 × per week, 50–70% HRres, Duration: 10 weeks	↑ FMD (p < 0.01) ∆ T = 5.0 ∆ C = -0.5	↓ SBP ↓ DBP ↑ VO_2_ peak ↓ % BF ↓ WC ↓ Weight ↓ BMI
Bhutani et al. (2013)^39^	Total = 40 Women = 38, Men = 2 T (n = 24): 42 years C (n = 16): 49 years	T: 35.0 ± 1.0 C: 35.0 ± 1.0	T: 6.8 ± 1.3 C: 7.0 ± 3.3	Aerobic, 20 a 40 min, 3 × per week, 60–75% HRmax, Duration: 12 weeks	↑ FMD (p < 0.05) ∆ T = 0.4 ∆ C = -0.7	↔ SBP ↔ DBP ↓ Weight ↔ Fat mass (kg) ↓ WC ↔ Leptin ↔ Adiponectin
Franklin et al. (2015)^38^	Total = 18 Women = 18 T (n = 10): 50 years C (n = 8): 30 years	T: 34.2 ± 3.0 C: 32.2 ± 6.9	T: 9.5 ± 1.6 C: 8.4 ± 3.5	Resistance Training, NI, 2 × week, 3 × 80–90% (1MR), Duration: 8 weeks	↑ FMD (p < 0.01) ∆ T = 0.3 ∆ C = -0.4	↔ SBP ↔ DBP ↑ VO_2_ peak ↓ Glucose ↓ % BF ↔ Weight ↔ CRP ↔ TNF-alpha
Bircher et al. (2007)^37^	Total = 26 Women = 13, Men = 13 T (n = 13): 30 years C (n = 13): 30 anos	T: 33.0 ± 3.1 C: 34.8 ± 3.6	T: 4.3 ± 3.5 C: 4.9 ± 2.9	Aerobic, 45 min, 3 × per weeks, 70% HRmax, Duration: 12 weeks	↑ FMD (p = 0.019) ∆ T = 2.6 ∆ C = -0.2	↔ SBP ↔ DBP ↑ VO_2_ peak ↓ % BF ↓ Weight
Olson et al. (2006)^34^	Total = 30 Women = 30 T (n = 15): 38 years C (n = 15): 38 years	T: 27.0 ± 5.0 C: 27.0 ± 6.0	T: 6.3 ± 2.7 C: 6.5 ± 2.3	Resistance Training, NI, 2 × per week, 3 × 8–10 MR, duration: 48 weeks	↑ FMD (p = 0.002) ∆ T = 2.6 ∆ C = -1.4	↓ SBP ↓ DBP ↓ % BF ↔ Weight ↔ EID
Robinson et al. (2016)^36^	Total = 19 Women = 14, Men = 5 T (n = 10): 34 years C (n = 9): 28 years	T: 33.0 ± 6.0 C: 32.0 ± 5.0	T: 9.3 ± 4.2 C: 8.6 ± 4.8	Aerobic, 30 to 45 min, 3 × week, 75% HRmax, Duration: 8 weeks	↓ FMD (p < 0.05) ∆ T = -0.9 ∆ C = 0.0	↓ SBP ↓ DBP ↓ % BF, ↓HR ↓ Weight ↔ EID ↔ TNF-alpha
Swift et al.* (*2012)^35^	Total = 91 Women = 91 T (n = 68): 57 years C (n = 23): 56 years	T: 31.5 ± 3.1 C: 32.0 ± 3.0	T: 4.0 ± 2.6 C: 4.7 ± 2.4	Aerobic, Energy Expenditure 4 kcal/kg, 3–4 × per week, 50% VO_2_ peak, Duration: 24 weeks	↑ FMD (p < 0.01) ∆ T = 1.0 ∆ C = -0.5	↔ SBP ↔ DBP ↔ VO_2_ peak
Swift et al. (2012)^35^	Total = 55 Women = 55 T (n = 32): 56 years C (n = 23): 57 years	T: 32.3 ± 4.3 C: 32.0 ± 3.0	T: 3.7 ± 2.6 C: 4.7 ± 2.4	Aerobic, Energy Expenditure 8 kcal/kg, 3–4 × per week, 50% VO_2_ peak, Duration: 24 weeks	↑ FMD (p < 0.01) ∆ T = 1.5 ∆ C = -0.5	↔ SBP ↔ DBP ↑ VO_2_ peak
Swift et al. (2012)^35^	Total = 55 Women = 55 T (n = 32): 56 years C (n = 23): 57 years	T: 30.7 ± 3.1 C: 32.0 ± 3.0	T: 4.4 ± 2.4 C: 4.7 ± 2.4	Aerobic, Energy Expenditure 12 kcal/kg, 3–4 × per week, 50% VO_2_ peak, Duration: 24 weeks	↑ FMD (p < 0.01) ∆ T = 1.2 ∆ C = -0.5	↔ SBP ↔ DBP ↑ VO_2_ peak
Tucker et al. (2021)^43^	Total = 27 Men = 27 T (n = 8): 29 years C (n = 9): 28 years	T: 29.7 ± 4.5 C: 29.6 ± 3.9	T: 6.2 ± 2.55 C: 4.7 ± 2.40	Aerobic,30 to 45 min, 4 × per week, 50% VO2max, Duration: 4 weeks	↔ FMD (p < 0.05) ∆ T = -0.7 ∆ C = -1.00	↔ SBP ↔ DBP ↑ VO_2_ max
Tuckeret al. (2021)^43^	Total = 27 Men = 27 T (n = 10): 30 years C (n = 9): 28 years	T: 30.2 ± 3.0 C: 29.6 ± 3.9	T: 6.0 ± 2.53 C: 4.7 ± 2.40	HIIT, 8 to 11 min, 4 × per week, 90 to 95% HRmax, Duration: 4 weeks	↔ FMD (p < 0.05) ∆ T =—0.5 ∆ C =—1.00	↔ SBP ↔ DBP ↑ VO_2_ max
Vélez-Ramírez et al. (2020)^42^	Total = 57 Women = 34, Men = 23 Age: 40.8 ± 7.06 years T (n = 14): ? years C (n = 16): ? y ears	30.04 ± 3.49	T: 12.89 ± 8.83 C: 10.48 ± 7.15	HIIT, 4 × 4 min, 3 × per week, 85 to 95% HRmax, Duration: 12 weeks	↔ FMD (p < 0.01) ∆ T = 2.79 ∆ C = 2.14	↑ VO_2_ max ↔ HRrest ↓BF% ↔ PWV
Vélez-Ramírez et al. (2020)^42^	Total = 57 Women = 34, Men = 23 Age: 40.8 ± 7.06 years T (n = 12): ? years C (n = 16): ? years	30.04 ± 3.49	T: 6.94 ± 4.22 C: 10.48 ± 7.15	Resistance training, 3 × per week, 30 to 40 min, 50 to 70% 1RM, Duration: 12 weeks	↑ FMD (p < 0.01) ∆ T = 10.54 ∆ C = 2.14	↑ VO_2_ max ↓ HRrest ↓ BF% ↑ PWV
Hovsepian et al. (2021)^41^	Total = 26 Women = 26 T (n = 13): 20.23 years C (n = 13): 20.69 years	T: 29.6 C: 32.34	T: 8.83 ± 3.71 C: 9.27 ± 5.01	HIIT, 4 × per week, 40 min, 85 to 90% HRmax, Duration: 10 weeks	↑ FMD (p < 0.01) ∆ T = 1.38 ∆ C = -1,20	↑ VO_2_ max ↓ BF% ↓ Weight

*FMD* flow-mediated dilation, *SBP* systolic blood pressure, *DBP* diastolic blood pressure, *HRmax* maximum heart rate, *HRres* heart rate reserve, *VO*_*2*_* peak* peak rate of oxygen consumption, *VO*_*2*_* max* maximum oxygen consumption, *T* training group, *C* control group, *HR* heart rate, *INA* intensity not available, *% BF* percentage of body fat, *WC* waist circumference (cm), *TNF-alpha* tumor necrosis factor-alpha, *CRP* C-reactive protein, *EID* endothelial-independent dilation of the brachial artery.

### Inclusion and exclusion criteria

The reviewers (M.B. and R.S.N.S.) applied the defined inclusion and exclusion criteria either manually or automatically using EndNote X9 software. The inclusion criteria were adults (≥ 18 years of age) with overweight and obesity; intervention duration of ≥ 4 weeks of aerobic or resistance training; and endothelial function as measured by FMD. The exclusion criteria were studies with animals or children/adolescents; sample with eutrophic individuals only (BMI ≤ 24.99 kg/m_2_); target population of the study described to be with diabetes, hypertension or metabolic syndrome; dietary and/or drug interventions; duplicate studies; and non-randomized and/or uncontrolled design (clinical trials, protocols and reviews).

### Data extraction and management

Two reviewers (M.B. and G.W.) independently read the full texts of all potentially eligible studies. For the studies deemed relevant to be included in the review, data were extracted manually using a pre-structured database form in Windows Office Excel 2010. GetDate Graph Digitizer (version 2.26) was used to extract the data from graphs. Three different sets of data were extracted:

1. study-related (author, journal, year of publication and FITT of exercise);
2. participants’ characteristics (age, gender, BMI and condition);
3. methods (randomization, blinding);
4. outcomes (sample size, means and measures of dispersion at baseline and post-intervention).

### Risk of individual bias

We rated the risk of bias of eligible studies using Cochrane Risk of Bias 2 (RoB 2) assessment tool^28^. This tool consists of six domains: (1) randomization process; (2) deviations from intended interventions—allocation concealment; (3) missing of outcome data; (4) measurement of the result; (5) selection of reported result; (6) absolute bias. From the risk of bias assessment, the studies selected are rated as: low risk of bias (in all domains); some concerns (in at least one domain for this result, but not high risk of bias in any domain); or high risk of bias (in at least one domain for this result or the study is judged to be at some concerns for multiple domains in a way that substantially reduces confidence in the outcome). The intervention effect or 'intention to treat' was the effect of interest used. No study was excluded based on the risk of bias assessment^28^.

### Certainty of evidence and strength of the recommendations

**Figure 2:**
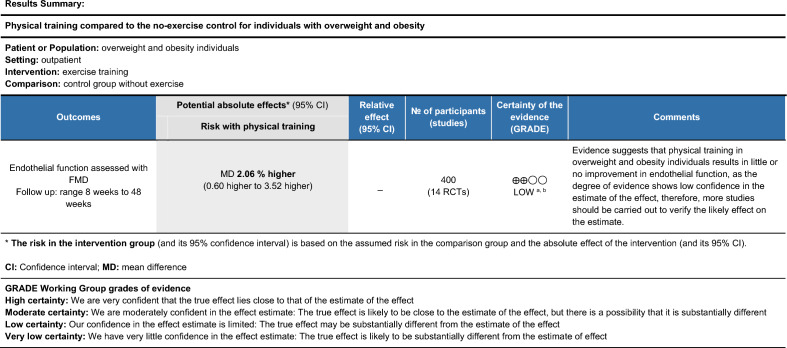
Quality of the body of evidence (GRADE quality rating) for the effect of exercise training in individuals with overweight and obesity.

**Figure 3:**
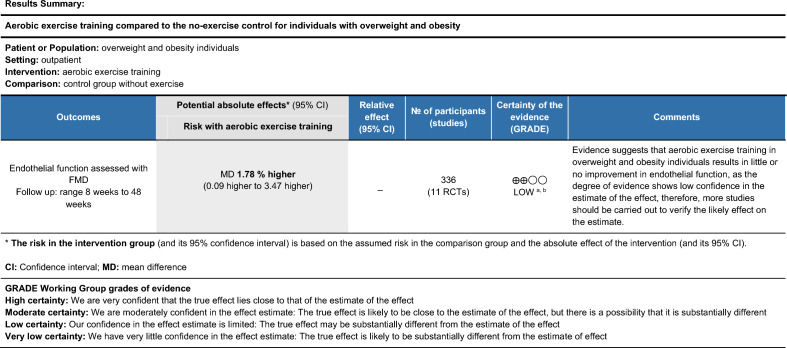
Quality of the body of evidence (GRADE quality rating) for the effect of aerobic training in individuals with overweight and obesity.

**Figure 4:**
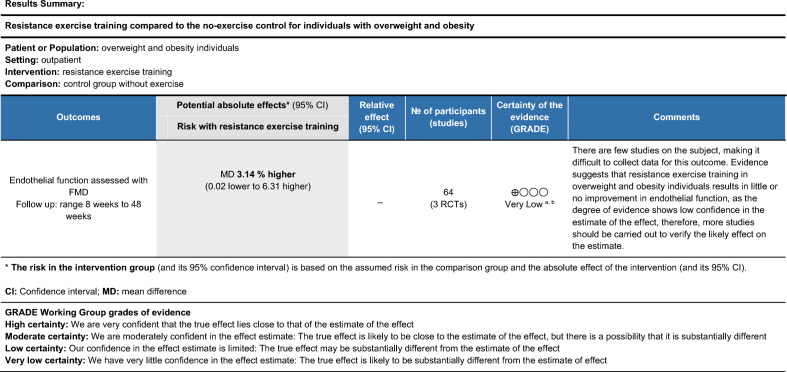
Quality of the body of evidence (GRADE quality rating) for the effect of resistance training in individuals with overweight and obesity.

To rank the strength of the body of evidence, we used the GRADE tool (Grading Recommendations, Rating, Development, and Evaluation)^25,29^ (Figs. 2, 3 and 4). GRADE ranks the quality of evidence into four levels: (1) high; (2) moderate; (3) low; (4) very low. This classification is based on the evaluation of confidence in five domains specific estimative: (1) methodological limitations (risk of bias); (2) inconsistency; (3) indirect nature of the evidence; (4) inaccuracy; (5) publication bias.

### Statistical analysis

We conducted analyses to determine the effect of aerobic or resistance training (G.W.). Effect measures were presented as mean differences (MDs) between the training group vs. no-exercise control group and related 95% confidence intervals (95% CIs). If the standard deviation (SD) of differences (post–pre) was not available in an eligible study, it was imputed from SDs at each time point (pre- and post-) together with a correlation coefficient of 0.5^25,30^: ▲SD = √ SD2 baseline + SD2 final − (2 × 0.5 × SD baseline × SD final).

Since the studies did not show enough similarities to warrant a fixed-effects model, all MDs were pooled using a random-effects model. Since 95% CIs are not the most suitable estimates for making decisions based on results, we considered the 95% prediction interval estimates (95% PI)^31^ as they reflect the effects to be expected in future studies, similar to the characteristics of the RCTs included in the meta-analysis. These estimates also reflect the variation of potential effects (harmful or beneficial) that are useful for clinical decision-making^31^. To avoid unit-of-analysis error for RCTs with multiple treatment arms and a single control group, the sample size of the control group was weighed by the number of groups and participants treated^17^. Heterogeneity (percentage of variability in effect estimates) was assessed by I^2^ statistic for each pairwise comparison as suggested by Higgins (0–40%: might not be important; 30–60%: may represent moderate heterogeneity; 50–90%: may represent substantial heterogeneity; 75–100%: considerable heterogeneity)^25,32^. Heterogeneity (p < 0.05) was tested using subgroup or meta-regression analyses for normally distributed effect modifiers in QQ-plots and verified by Shapiro Wilk’s test^32^. In order to remove discrepant data from the meta-analysis, the inaccuracy of effect estimates due to heterogeneity was also be visually identified when their 95% CIs failed to overlap in the forest plots^32^*.* Potential effect modifiers, including age, baseline FMD (intervention and control groups), baseline delta FMD values (intervention vs. control groups) and FITT components were analyzed separately. We performed the Egger's test via a funnel plot to assess publication bias (10 or more studies)^33^. All statistical tests were two-tailed and the level of significance was set at p < 0.05. The measures of dispersion expressed as CIs or standard errors were converted into standard deviations (SDs** = **EP × √n) prior to the analysis. Data models were built in RStudio software (version 1.3.959) using R package meta (version 3.6.1) for Windows (https://www.r-project.org/). Supplementary Table 2 shows the main RStudio script (supplementary material).

## Results

### Description of the selected studies

A total of 2471 studies were identified through our search strategy (March 2022). All duplicates were removed (208 studies) and 2,263 studies were excluded after screening their titles and/or abstracts. All 15,573 documents retrieved from searches in the gray literature and unpublished works were also excluded after reading the titles and/or abstracts. The remaining 17 studies were read in full. Of these, seven were excluded because they did not use a control group (n = 3), missing information (n = 2), no participants with overweight and obesity (n = 1), and not a RCT (n = 1). There remained 10 studies: three including overweight adults^34–36^, four adults with obesity^37–40^ and three adults with overweight and obesity^41–43^. They were all included in the quantitative synthesis. Figure 1 shows a detailed description.

As for the intervention, five studies used continuous aerobic training^35–37,39,40^, two resistance training^34,38^, one high-intensity interval training (HITT)^41^, one used both HITT and resistance training^42^, and one used continuous aerobic training and HITT^43^. Table 1 details the study interventions.

The quantitative analysis included 400 individuals with overweight (157 in the intervention group and 47 in the no-exercise control group) and obesity (59 in the intervention group and 49 no-exercise control group), and overweight + obesity (57 in the intervention group and 38 no-exercise control group). The participants’ age ranged from 20 to 57 years (mean 42 years) in the intervention groups and 20 to 56 years (mean 41 years) in the no-exercise groups. Mean baseline FMD values were 6.9 ± 2.3% in the intervention groups and 6.4 ± 2.7% in the no-exercise groups. Of the 10 studies selected, five were women only^34,35,38,40,41^, one included men only^43^ and four included both female and male adults^36,37,39,42^. Information on male-to-female ratio in each group was not available for one study^37^. There was a higher proportion of women (n = 318) than men (n = 89) among the participants.

Three studies involved overweight participants^34–36^ and four involved participants with obesity^37–40^ and associated comorbidities. The most common comorbidities were hypertension^35,40^ and type 2 diabetes^37^ seen in nearly 40% of the sample. Of the 10 studies eligible for the meta-analysis, nine were in English^34–36,38–43^ and one was in German^37^.

Of the 10 studies included in this review, three of them reported FMD after upper arm occlusion^34,37,42^. The effects of the menstrual cycle phase on FMD^44,45^ were taken into account in only two studies^34,38^. As for food intake and daily level of physical activity during the intervention period, daily energy requirements were reported in one study^43^. Participants were asked to maintain their daily life routines during the study intervention in four studies^34,37,40,41^ and a healthy lifestyle brochure was handed out to participants in two studies^38,42^. Information on diet and physical activity was not available in four studies^35,36,41,43^.

### Methodological quality

**Figure 5:**
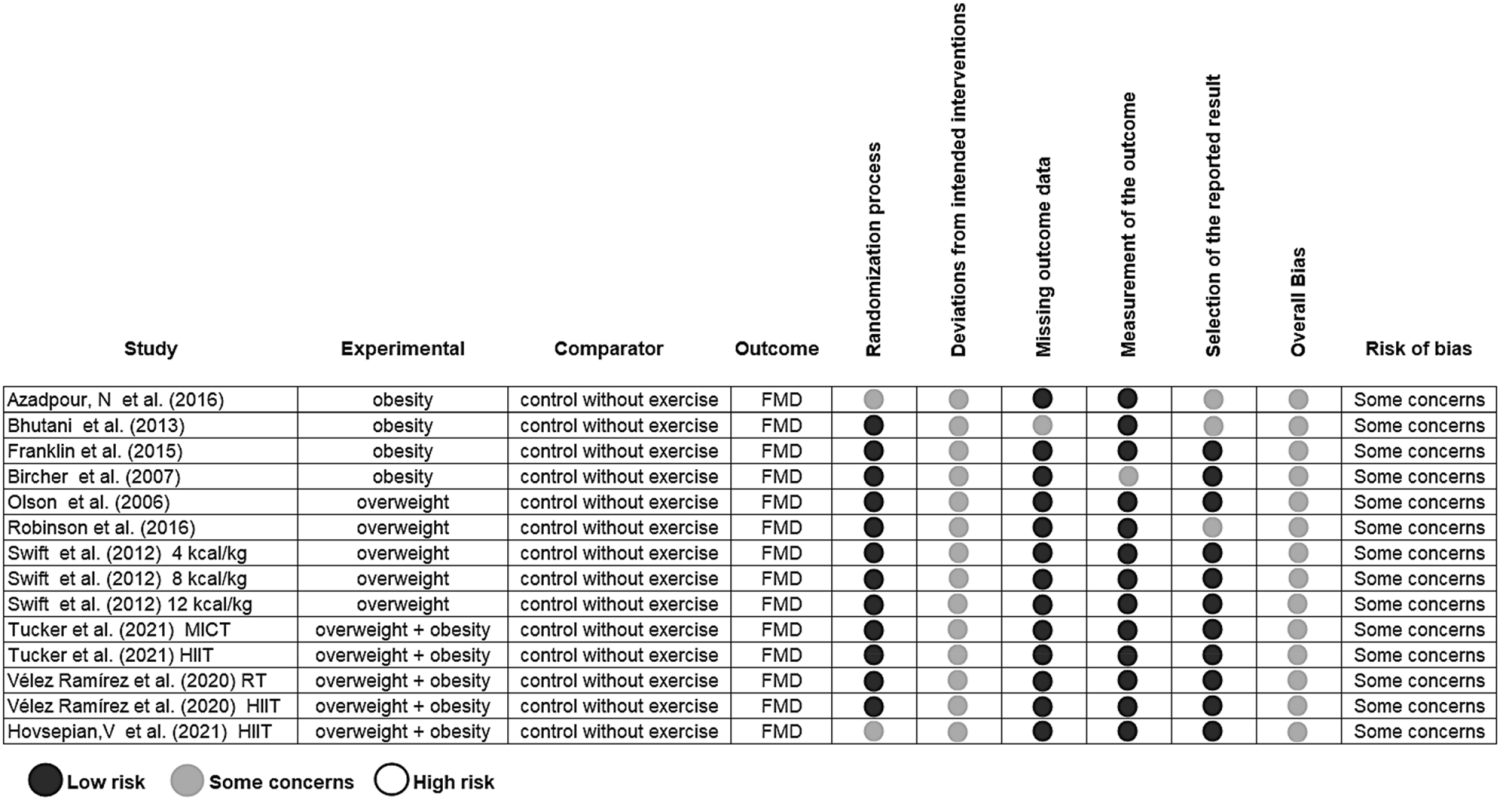
Risk of bias assessment using Cochrane’s risk-of-bias 2 (RoB 2) tool. FMD, flow-mediated dilation.

Twelve of the fourteen groups reviewed were rated as at low risk and two was judged as raising some concerns of *bias arising from the randomization process* using RoB 2 tool. All groups were judged as raising some concerns of *bias due to deviations from the intended intervention* because blinding is not possible for participants of exercise interventions. Thirteen groups were rated as at low risk and one as raising some concerns of *bias due to missing outcome data*. Thirteen groups were rated at low risk and one as raising some concerns of *bias due to measurement of the outcome*. And eleven groups were rated as at low risk and three as raising some concerns of *bias arising from selection of the reported result*. All studies reported intention-to-treat analyses and point estimates of the effect size. Together all fourteen groups included in this meta-analysis were rated as raising some concerns for the risk of bias (Fig. 5)**.**

The quality of the body of evidence from all studies included in this meta-analysis was rated as high using the GRADE approach due to their randomized controlled design^29^. However, we applied a one-point reduction in the risk of bias domain for exercise training in general and aerobic training since blinding of participants to exercise intervention is not feasible. We also applied one-point reduction in the inconsistency domain due to different sample characteristics and small number of studies, they were rated as low confidence.

We applied a one-point reduction in resistance training for risk of bias, inconsistency and imprecision. The imprecision domain showed a wide confidence interval indicating uncertainty of the evidence from a small number of studies. We thus considered very low certainty of the body of evidence for resistance training.

### Description of exercise training sessions

Table 1 describes the individual studies included in this meta-analysis. Though all studies reported supervised exercise sessions, sessions were only supervised over 16 out of 48 weeks of training in one study^34^.

The frequency of exercise training ranged from 2 to 4 days per week. The mean duration was 18 weeks, ranging from 8 to 48 weeks. Aerobic exercise was most often prescribed at moderate intensity (60–75% of maximum heart rate, HRmax)^36,37,39,42,43^ and at high intensity in three studies (85–90 to 90–95% of HRmax)^41–43^. For prescribing moderate-intensity exercise, one study reported using HR reserve (50–70%)^40^, another one reported using maximum oxygen consumption (50% VO_2_max)^43^ and another one reported using peak oxygen consumption (50% VO2peak)^35^. The one-repetition maximum test (1-RM) was used to set the intensity of resistance exercise (50–70% of 1-RM for low to moderate intensity or 80–90% of 1-RM for moderate to high intensity)^38,42^ in two studies and 8–10 repetition maximum was used in another one^34^.

Aerobic training sessions lasted 25–50 min. The duration of resistance exercise sessions was not available in two studies^34,38^. Resistance training volume was reported as the number of sets and repetitions per session in two studies^34,38^ and as energy expenditure in another one^42^.

As for type/modality of exercise, aerobic training consisted of continuous walking or running on a treadmill^36,37,40,42^, exercising on a cycle ergometer^39,41,43^, or a mix of both^35^. The resistance training session involved a sequence of exercises performed on a machine in one study^38^, a mix of exercising with free weights and exercising on a machine in one study^34^, and no information was available in another study^42^. The participants exercised both upper and lower limbs and the number of sets of exercise ranged from 6 to 10 per session^34,38,42^.

### Effect size of exercise training on FMD

**Figure 6:**
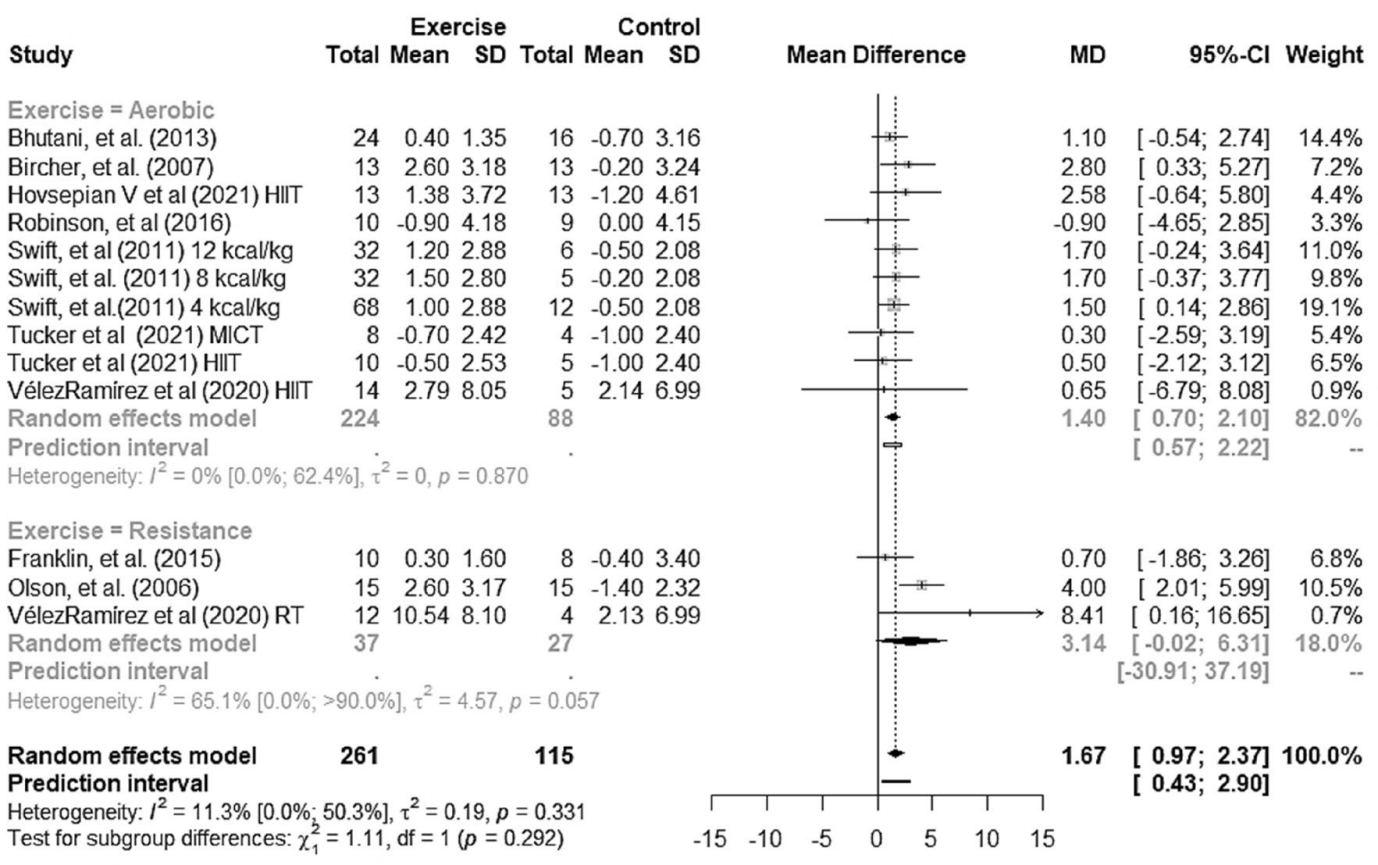
Forest plot for the pooled mean effect of exercise training and for subgroup (aerobic, resistance) versus no-exercise in individuals with overweight and obesity. *HITT* high-intensity interval training, *MICT* moderate-intensity continuous training, *RT* resistance training.

Exercise training improved FMD (1.67% [95% CI 0.97–2.37]), p < 0.001) in adults with overweight and obesity compared to no-exercise controls. In addition, the 95% PI, i.e., the range of uncertainty of this result expected in future RCTs, was 0.43–2.90% (Fig. 6). When we compared exercise training modalities in individuals with overweight and obesity, we found improved FMD for aerobic training (1.40% [95% CI + 0.70 to + 2.10], p < 0.001, 95% PI + 0.57 to + 2.22,) but not for resistance training (3.14% [95% CI + 0.02 to + 6.31], p = 0.051, 95% PI − 30.91 to 37.19) (Fig. 6).

**Figure 7:**
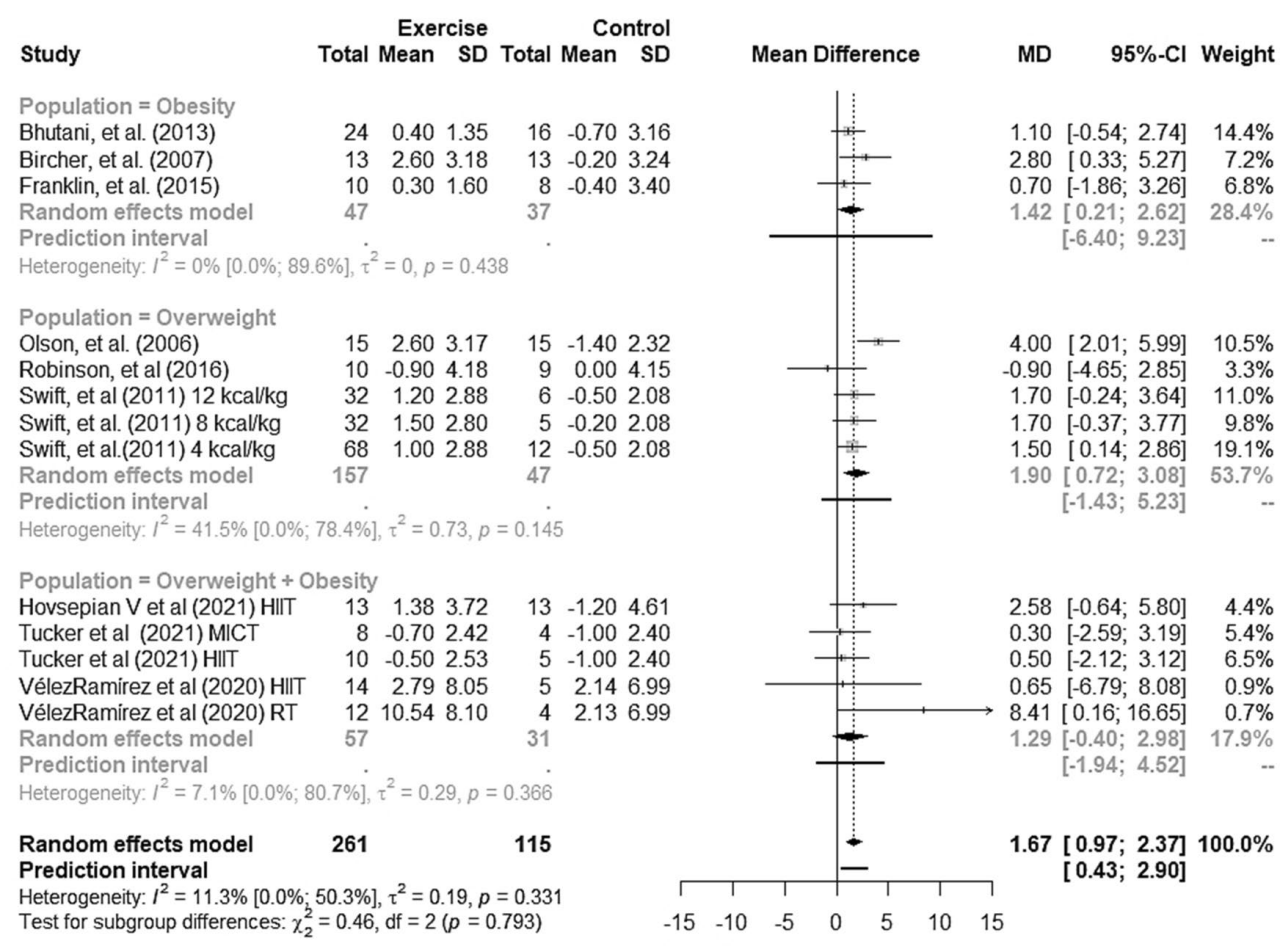
Forest plot for the pooled mean effect of exercise training and for subgroup (overweight, obesity and overweight + obesity) versus no-exercise in individuals with overweight and obesity. *HITT* high-intensity interval training, *MICT* moderate-intensity continuous training, *RT* resistance training.

The analysis of the results in adults with overweight and obesity by BMI groups did not show any changes in FMD (overweight, 1.90% [95% CI + 0.72 to + 3.08], p = 0.002, 95% PI − 1.43 to + 5.23, obesity, 1.42% [95% CI − 0.21 to + 2.62%], p = 0.021, 95% PI − 6.40 to + 9.23, overweight + obesity, 1.29% [95% CI − 0.040 to + 2.98], p = 0.134, 95% PI − 1.94 to + 4.52), with no differences between the groups (p = 0.793) (Fig. 7).

The measures of heterogeneity using Cochrane Q and I^2^ statistics in the forest plots indicated that heterogeneity of effect sizes for exercise training (I^2^ 11.3%, p = 0.331) and aerobic training (I^2^ 0.0%, p = 0.870) “might not be important”, but may represent ‘’substantial heterogeneity" for resistance training (I^2^ 65.1%, p = 0.057), which further supports our choice of using a random-effects model. Of the studies initially included in the analysis, Azadpour et al.^40^ study was the one that contributed most to heterogeneity (Fig. 2s). As the CIs did not overlap, we then decided to exclude its data from the meta-analysis. For the sake of transparency, the analysis including data from Azadpour et al.^40^ study is presented in the supplementary material (Supplementary Figs. 1–5).

### Moderator variables and regression analysis

Potential effect moderator variables, including number of exercise sessions (total frequency of exercise), age (intervention and control groups), baseline FMD (intervention and control groups), and baseline delta FMD values (intervention vs. control groups), were analyzed using meta-regression. For pooled data from exercise training in participants with overweight and obesity, none of the potential moderators tested showed any interference with the summary results (Supplementary Figs. 2–8).

### Publication bias

Given the sufficient number of groups (n = 13) included in this meta-analysis, we performed a linear regression analysis using a funnel plot. When the number of studies included in the meta-analysis is smaller than 10, the funnel plot test has low power to detect publication bias. The results showed no publication bias (Egger's test, p = 0.815)^46^. The funnel plot can be found in the supplementary material (Supplementary Fig. 9).

## Discussion

In this systemic review with meta-analysis, we found clinically relevant (> 1%) improvement in endothelial function as measured by FMD in response to exercise. Despite significant results for aerobic training, the evidence for resistance training was not sufficient to reject the null hypothesis (95% CI − 0.02 to 6.31). Thus, we cannot conclusively state that resistance training is superior when compared to no-exercise controls in overweight individuals. When we analyzed the participants by BMI groups, we found a similar response in overweight, obese and overweight + obesity groups. To the best of our knowledge, this is the first meta-analysis to evaluate the effect of exercise training on FMD in adults with overweight and obesity.

Current evidence has shown endothelial dysfunction in individuals with overweight and obesity (BMI ≥ 25.0 kg/m^2^)^6^. The benefits of exercise training on FMD in this population were reported in a structured meta-analysis by Son et al.^47^. Yet, their analysis included studies with heterogeneous populations selected based on their health status (heart failure, metabolic syndrome, type 2 diabetes and “healthy”). So, a comparison of our data with those from Son et al.^47^ would yield discrepant information since our analysis included studies of adults with overweight and obesity as target population.

The recommended management for reducing BMI and/or visceral adiposity involve higher levels of daily physical activity associated with a healthy balanced diet^48^. These recommendations can also promote improvement in FMD in individuals with overweight and obesity and cardiovascular risk factors such as type 2 diabetes^49^ and arterial hypertension^17^, as previously demonstrated by our group.

Since various factors may play a role, including changes in BMI, visceral adiposity, lipid profile, fasting glucose and blood pressure, it is difficult to establish a cause-effect relationship between exercise training response and improvement in endothelial function in individuals with overweight and obesity^50^. Moreover, the differences in these factors between exercise training and no-exercise groups have been little explored in the RCTs included in this meta-analysis, which further limits our ability to draw inferences. Still, considering that we chose to exclude from this meta-analysis studies primarily including individuals with medical conditions such as diabetes, hypertension or metabolic syndrome, we believe that the finding of an effect on FMD is largely due to vascular endothelial response to exercise training in the population studied^51^.

Several modifiable factors of exercise training that are associated with overweight and obesity may also in part explain FMD findings in this meta-analysis, including angiotensin II (a potent vasoconstrictor)^52^, lipid profile (associated with vascular damage)^53,54^, sympathetic activity (inducing strong vasoconstriction)^55^, levels of leptin (a peptide hormone that inhibits NO)^52^ and blood pressure levels (causing damage and vascular remodeling)^54^. These factors lipid profile^34,36,38^, blood pressure^34–36,38,39^, fast glucose^34,36,38^ and leptin^39^ were not fully explored in the RCTs included in this meta-analysis so we could not conduct a meta-regression and prevented further discussions.

We found low heterogeneity of studies in this meta-analysis. We did not find significant results for resistance training (p = 0.051), which may be due to the low number of studies included^56^. In addition, we performed subgroup analysis, removed discrepant data (no overlapping 95% CIs) and conducted a meta-regression to explain it^56^. A subgroup analysis for exercise modality (aerobic or resistance) showed improvement in FMD only in response to aerobic training in individuals with overweight and obesity. It is worth noting that only three RCTs involving resistance training met the inclusion criteria for this meta-analysis (18.0% of the weight data analyzed). Therefore, the effect of resistance training found should be interpreted with caution.

As for BMI groups (overweight, obesity or overweight + obesity), the effect estimate in the subgroup analysis was similar in the groups. However, removing data from Azadpour et al. (2016) study^40^ together with a meta-regression analysis of difference in baseline FMD between the intervention and control group largely explained the heterogeneity observed.

Of the RCTs selected, Robinson et al.^36^ and Tucker et al.^43^ did not report an effect of aerobic training on FMD. A potential explanation for this null-finding could be intervention duration of only ≤ 8 weeks^36,43^. A previous meta-analysis pointed to a positive correlation between training duration (weeks) and improvement in FMD though it included heterogeneous populations^47^.

Another interesting finding described by our meta-regression was that none of the potential moderators showed interference with the summary results. An inverse correlation between baseline FMD and response to exercise is reported in the literature^57^. In addition, it shows that FMD data may be underestimated in different interventions involving individuals with intact endothelial function or large baseline variation.

We added 95% PI estimates to our results as they reflect highly likely values for the true effects of exercise training on FMD in future RCTs^58^. The 95% PIs ranged from 0.43 to 2.90% for FMD response to exercise training, 0.57–2.22 for aerobic training, which was not conclusive for resistance training (− 30.91 to + 37.19). The slope indicating the same direction of the effect found in this meta-analysis is an important finding, which supports the relevance of our results especially for aerobic training.

The dispersion of the pooled sample means for the effect of aerobic training on FMD (95% CI + 0.70 to + 2.10%) shows small variation and further supports the effect estimate found. Although our findings indicate that overall, the aerobic training intervention led to an improvement in FMD in individuals with overweight and obesity, more studies are necessary to further explore the effect of resistance training on FMD. The limitations of the present study include relatively small sample sizes that limit the ability to control for all comorbidities that might confound the results, relatively short duration of exercise training, and small number of RCTs that met our inclusion criteria, in particular those including participants with overweight and obesity and no other medical conditions. It is yet unclear what type of aerobic training is more effective in improving endothelial function.

Also, it is important to mention studies with different strategies of exercise training evidencing promising results for cardiometabolic health in overweight and obese population. In this sense, the well conducted systematic review and networking metanalysis conducted by Batrakoulis et al.^59^, including 81 randomized controlled trials (4331 participants), and involving exercise interventions consisting of continuous endurance training, interval training, resistance training, combined aerobic and resistance training, and hybrid-type training, demonstrated that mainly combined training was able to improve variables like body composition, lipid metabolism, glucose control, blood pressure, cardiorespiratory fitness and muscular strength, in this population. However, FMD wasn’t evaluated in these studies.

Considering the potential of aerobic training to provide a benefit in FMD found together with the steep slope of PI in the same direction, we believe that our results are close to the actual effect of either aerobic or resistance exercise training on FMD in this population. Yet, other RCTs involving FMD and different modalities of exercise training in individuals with overweight and obesity are needed to support our findings.

## Conclusions

This systematic review and meta-analysis showed that exercise training leads to improvement in brachial artery FMD in individuals with overweight and obesity and this is a clinically relevant effect. Therefore, our findings demonstrate that exercise training is highly recommended for the promotion of cardiovascular health of individuals with overweight and obesity. However, the majority of the RCTs included in our study adopted aerobic training as intervention. Thus, more studies are needed evaluating the effects of strength modalities and the combination of resistance training and aerobic training on the FMD of individuals with overweight and obesity. Additionally, FMD improvement appears to be independent of BMI groups and dependent on the modality of exercise training. This finding should be interpreted with caution because of small number the studies included in our review.

## Supplementary Information

Supplementary Information.

## Data Availability

For more information, please contact the correspondent author. Correspondence and requests for materials should be addressed M.I.S.
