# Supplementary material for: Effect of aerobic and resistance exercise training on endothelial function in individuals with overweight and obesity: a systematic review with meta-analysis of randomized clinical trials
Source: Sci Rep. 2023 Jul 21;13:11826. doi: 10.1038/s41598-023-38603-x (PMC10362025; doi:10.1038/s41598-023-38603-x)
Supplement: Supplementary file 1 — Supplementary Information. [file 41598_2023_38603_MOESM1_ESM.docx]

Supplement material

Effect of Aerobic and Resistance Exercise Training on Endothelial Function in Individuals with Overweight and Obesity: A Systematic Review with Meta-Analysis of Randomized Clinical Trials

| MEDLINE (PubMed) |
| --- |
| *Overweight and Obesity : (Body Weight OR Diet, Reducing OR Skinfold Thickness OR Obesity OR Overweight OR Fats, Intra-Abdominal OR Intra Abdominal Fat OR Intra-Abdominal Fats OR Fat, Intra-Abdominal OR Fat, Intra Abdominal OR Intra-Abdominal Adipose Tissue OR Adipose Tissue, Intra-Abdominal OR Intra Abdominal Adipose Tissue OR Retroperitoneal Fat OR Fat, Retroperitoneal OR Fats, Retroperitoneal OR Retroperitoneal Fats OR Retroperitoneal Adipose Tissue OR Adipose Tissue, Retroperitoneal OR Visceral Fat OR Fat, Visceral OR Fats, Visceral OR Visceral Fats OR Abdominal Visceral Fat OR Abdominal Visceral Fats OR Fat, Abdominal Visceral OR Fats, Abdominal Visceral OR Visceral Adipose Tissue OR Adipose Tissue, Visceral) AND*  *Exercise: (exercise OR exercises, isometric OR isometric exercises OR warm-up exercise OR exercise, warm-up OR exercises, warm-up OR warm up exercise OR warm-up exercises OR exercise, aerobic OR aerobic exercises OR exercises, aerobic OR aerobic exercise OR endurance, physical OR endurance, physical OR physical endurance OR training, resistance OR strength training OR training, strength OR weight-lifting OR strengthening program OR strengthening program, weight-lifting OR strengthening programs, weight-lifting OR weight lifting strengthening program OR weight-lifting strengthening programs OR weight-lifting exercise program OR exercise program, weight-lifting OR exercise programs, weight-lifting OR weight lifting exercise program OR weight-lifting exercise programs OR weight-bearing strengthening program OR strengthening program, weight-bearing OR strengthening programs, weight-bearing OR weight bearing strengthening program OR weight-bearing strengthening programs OR weight-bearing exercise program OR exercise program, weight-bearing OR exercise programs, weight-bearing OR weight bearing exercise program OR weight-bearing exercise programs OR activities, motor OR activity, motor OR motor activities OR physical activity OR activities, physical OR activity, physical OR physical activities OR locomotor activity OR activities, locomotor OR activity, locomotor OR locomotor activities) AND*  *Endothelium:* (*Vascular Endothelium OR Endotheliums, Vascular OR Vascular Endotheliums OR Capillary Endothelium OR Capillary Endotheliums OR Endothelium, Capillary OR Endotheliums, Capillary OR Vasorelaxation OR Vasodilatation OR Vascular Endothelium-Dependent Relaxation OR Endothelium-Dependent Relaxation, Vascular OR Relaxation, Vascular Endothelium-Dependent OR Vascular Endothelium Dependent Relaxation OR hyperemia OR reactive hyperemia OR hyperemia, reactive OR hyperemia, reactive OR reactive hyperemia OR active hyperemia OR hyperemia, active OR arterial hyperemia OR hyperemia, arterial OR venous engorgement OR engorgement, venous OR venous congestion OR congestion, venous OR passive hyperemia OR hyperemia, passive OR flow-mediated dilation OR plethysmography) AND*  *Type of stady: (Randomized controlled trial[pt] OR controlled clinical trial[pt] OR randomized controlled trials[mh] OR random allocation[mh] OR double-blind method[mh] OR single-blind method[mh] OR clinical trial[pt] OR clinical trials[mh] OR (“clinical trial”[tw]) OR ((singl*[tw] OR doubl*[tw] OR trebl*[tw] OR tripl*[tw]) AND (mask*[tw] OR blind*[tw])) OR (“latin square”[tw]) OR placebos[mh] OR placebo*[tw] OR random*[tw] OR research design[mh:noexp] OR follow-up studies[mh] OR prospective studies[mh] OR cross-over studies[mh] OR control*[tw] OR prospectiv*[tw] OR volunteer*[tw])* |
| EMBASE |
| *Overweight and Obesity: (' Body Weight'/exp OR ‘Overweight’ OR 'obesity')AND*  *Exercise training: ('exercise' OR 'aerobic exercise' OR 'isometric exercise' OR 'warm-up exercise' OR 'physical endurance' OR 'endurance' OR 'training' OR 'resistance training' OR 'weight lifting' OR 'strengthening exercise' OR 'weight bearing' OR 'motor activity' OR 'physical activity') AND*  *Endothelium:('vascular Endothelium' OR 'capillary endothelium' OR ‘vasorelaxation’ OR ‘vasodilatation’ OR ‘vascular endothelium-dependent relaxation' OR ‘hyperemia’ OR ‘reactive hyperemia’ OR ‘arterial hyperemia’ OR ‘flow-mediated dilation’ OR ‘plethysmography’) AND*  *Type of stady: ('randomized controlled trial' OR 'controlled clinical trial')* |
| COCHRANE |
| *Overweight and Obesity : (Body Weight OR Diet, Reducing OR Skinfold Thickness OR Obesity OR Overweight OR Fats, Intra-Abdominal OR Intra Abdominal Fat OR Intra-Abdominal Fats OR Fat, Intra-Abdominal OR Fat, Intra Abdominal OR Intra-Abdominal Adipose Tissue OR Adipose Tissue, Intra-Abdominal OR Intra Abdominal Adipose Tissue OR Retroperitoneal Fat OR Fat, Retroperitoneal OR Fats, Retroperitoneal OR Retroperitoneal Fats OR Retroperitoneal Adipose Tissue OR Adipose Tissue, Retroperitoneal OR Visceral Fat OR Fat, Visceral OR Fats, Visceral OR Visceral Fats OR Abdominal Visceral Fat OR Abdominal Visceral Fats OR Fat, Abdominal Visceral OR Fats, Abdominal Visceral OR Visceral Adipose Tissue OR Adipose Tissue, Visceral) AND*  *Exercise training: (exercise OR exercises, isometric OR isometric exercises OR warm-up exercise OR exercise, warm-up OR exercises, warm-up OR warm up exercise OR warm-up exercises OR exercise, aerobic OR aerobic exercises OR exercises, aerobic OR aerobic exercise OR endurance, physical OR endurance, physical OR physical endurance OR training, resistance OR strength training OR training, strength OR weight-lifting OR strengthening program OR strengthening program, weight-lifting OR strengthening programs, weight-lifting OR weight lifting strengthening program OR weight-lifting strengthening programs OR weight-lifting exercise program OR exercise program, weight-lifting OR exercise programs, weight-lifting OR weight lifting exercise program OR weight-lifting exercise programs OR weight-bearing strengthening program OR strengthening program, weight-bearing OR strengthening programs, weight-bearing OR weight bearing strengthening program OR weight-bearing strengthening programs OR weight-bearing exercise program OR exercise program, weight-bearing OR exercise programs, weight-bearing OR weight bearing exercise program OR weight-bearing exercise programs OR activities, motor OR activity, motor OR motor activities OR physical activity OR activities, physical OR activity, physical OR physical activities OR locomotor activity OR activities, locomotor OR activity, locomotor OR locomotor activities) AND*  *Endothelium:* (*Vascular Endothelium OR Endotheliums, Vascular OR Vascular Endotheliums OR Capillary Endothelium OR Capillary Endotheliums OR Endothelium, Capillary OR Endotheliums, Capillary OR Vasorelaxation OR Vasodilatation OR Vascular Endothelium-Dependent Relaxation OR Endothelium-Dependent Relaxation, Vascular OR Relaxation, Vascular Endothelium-Dependent OR Vascular Endothelium Dependent Relaxation OR hyperemia OR reactive hyperemia OR hyperemia, reactive OR hyperemia, reactive OR reactive hyperemia OR active hyperemia OR hyperemia, active OR arterial hyperemia OR hyperemia, arterial OR venous engorgement OR engorgement, venous OR venous congestion OR congestion, venous OR passive hyperemia OR hyperemia, passive OR flow-mediated dilation OR plethysmography)* |
| LILACS |
| 1. English   *Overweight and Obesity : (obesity OR overweight) AND*  *Exercise training: (exercise OR exercise therapy OR physical exertion OR physical fitness OR motor activity) AND*  *Endothelium:* (*endothelium OR hyperemia OR endothelium, vascular OR endothelial cells)*   1. Portuguese (Brazil)   *Overweight and Obesity :(obesidade OR sobrepeso) AND*  *Exercise training: (exercício OR terapia com exercício OR esforço físico OR aptidão física OR atividade motora) AND*  *Endothelium: (endotélio OR hiperemia OR endotélio, células vasculares OR células endoteliais)*   1. Espanish   *Overweight and Obesity: (obesidad OR sobrepeso) AND*  *Exercise training: (ejercicio OR terapia con ejercicios OR esfuerzo físico OR aptitud física OR actividad motora) AND*  *Endothelium: (endotelio OR hiperemia OR endotelio, vascular OR células endoteliales)* |
| GRAY LITERATURE AND UNPUBLISHED STUDIES |
| *OpenGrey:endothelial AND exercise AND obesity OR overweight*  *Banco de Teses e Dissertações CAPES*: treinamento físico AND função endotelial AND sobrepeso OR obesos*  *ReBEC**: função endotelial (search 1), hiperemia reativa (search 2), dilatação mediada pelo fluxo (search 3)*  *ClinicalTrials***: overweigth OR obesity, flow-mediated dilation OR plethysmography OR reactive hyperemia OR vascular endothelium OR endothelium-dependent relaxation, exercise OR physical training OR physical activity*  *WHO: overweigth OR obesity, flow-mediated dilation OR plethysmography OR reactive hyperemia OR vascular endothelium OR endothelium-dependent relaxation, exercise OR physical training OR physical activity*  ** Filter: Grande Área Conhecimento (CIÊNCIAS DA SAÚDE), Área Avaliação (EDUCAÇÃO FÍSICA, ENFERMAGEM, MEDICINA I, MEDICINA II, MEDICINA III, NUTRIÇÃO)*  *** Filter: tipo de estudo (intervencional), Situação de recrutamento (Recrutando, Recrutamento concluído, Análise de dados complete)*  **** Filter: Stady type (interventional study (clinical trial))* |
| Supplement table 1. Details on gray and unpublished literature searches. |

| - library (readxl) - SobObe_Sem_Out <- read_excel ("SobObe_Sem_Out.xlsx") - View(SobObe_Sem_Out) - meta_FMD1= SobObe_Sem_Out <- metacont (t_n,t_mean,t_dp,c_n,c_mean,c_dp,Stady, byvar = Population, predict=TRUE, data = SobObe_Sem_Out, sm="MD") - meta_FMD1 - meta_FMD1= SobObe_Sem_Out <- metacont (t_n,t_mean,t_dp,c_n,c_mean,c_dp,Stady, predict=TRUE, data = SobObe_Sem_Out, sm="MD") - meta_FMD1 - forest (meta_FMD1, sortvar = Stady, xlim = c (-7.0, 15.0), predict= TRUE, col.square = "grey", col.diamond = "black", digits = 1) - forest(meta_FMD1,comb.fixed = FALSE, sortvar = Stady, xlim = c(-4.0, 9.0), digits.sd = 2, digits.I2= 2, digits.tau2 = 2, digits.pval.Q= 3, squaresize= 0.6, lab.e="Exercise training", lab.c="Control", col.inside = "black", col.square = "grey", col.diamond = "black",col.predict = "transparent", digits = 2) - baujat (meta_FMD1) - metainf (meta_FMD1, pooled= "random") - Freq_Total <- (c (16,36,16,96,24,72,72,72)) - qqnorm (Freq_Total) - qqline (Freq_Total,col = "Black", lwd = 2) - shapiro.test (Freq_Total) - metareg (meta_FMD1, ~Freq_Total) - meta_FMD1 <- metareg (meta_FMD1, ~Freq_Total) - bubble (meta_FMD1, col.line = "blue", col = "black", studlab = TRUE) - plot (Freq_Total, residuals(meta_FMD1)) - abline(h=0) - Age_c <- (c (42.0,30.8,50.1,38.0,28.0,56.8,56.8,56.8)) - qqnorm (Age_c, pch = 1) - qqline (Age_c, col = "Black", lwd = 2) - shapiro.test (Age_c) - metareg (meta_FMD1, ~Age_c) - meta_FMD1 <- metareg (meta_FMD1, ~Age_c) - bubble(meta_FMD1, col.line = "blue",col = "black", studlab = TRUE) - plot (Age_c, residuals(meta_FMD1)) - abline(h=0) - Age_t <- (c (49.0,30.8,51.8,38.0,34.0,57.4,55.9,56.3)) - qqnorm (Age_t, pch = 1) - qqline (Age_t,col = "Black", lwd = 2) - shapiro.test (Age_t) - metareg (meta_FMD1, ~Age_t) - meta_FMD1 <- metareg (meta_FMD1, ~Age_t) - bubble(meta_FMD1, col.line = "blue", col = "black", studlab = TRUE) - plot (Age_t, residuals(meta_FMD1)) - abline(h=0) - Baseline_FMD_c <- (c (6.8,9.5,4.3,3.4,8.6,4.0,4.4,3.7)) - qqnorm (Baseline_FMD_c, pch = 1) - qqline (Baseline_FMD_c, col = "Black", lwd = 2) - shapiro.test (Baseline_FMD_c) - metareg (meta_FMD1, ~Baseline_FMD_c) - meta_FMD1 <- metareg (meta_FMD1, ~Baseline_FMD_c) - bubble (meta_FMD1, col.line = "blue",col = "black", studlab = TRUE) - plot (Baseline_FMD_c, residuals(meta_FMD1)) - abline(h=0) - Baseline_FMD_t <- (c (7.0,8.4,4.9,6.3,9.3,4.7,4.7,4.7)) - qqnorm (Baseline_FMD_t, pch = 1) - qqline (Baseline_FMD_t, col = "Black", lwd = 2) - shapiro.test(Baseline_FMD_t) - metareg (meta_FMD1, ~Baseline_FMD_t) - meta_FMD1 <- metareg (meta_FMD1, ~Baseline_FMD_t) - bubble (meta_FMD1, col.line = "blue",col = "black", studlab = TRUE) - plot (Baseline_FMD_t, residuals(meta_FMD1)) - abline(h=0) - Delta_Baseline_FMD_t_c <- (c (0.2,-1.1,0.6,2.9,0.7,0.7,0.3,1.0)) - qqnorm (Delta_Baseline_FMD_t_c, pch = 1) - qqline (Delta_Baseline_FMD_t_c,col = "Black", lwd = 2) - shapiro.test (Delta_Baseline_FMD_t_c) - metareg (meta_FMD1, ~Delta_Baseline_FMD_t_c) - meta_FMD1 <- metareg (meta_FMD1, ~Delta_Baseline_FMD_t_c) - bubble (meta_FMD1, col.line = "blue",col = "black", studlab = TRUE) - plot (Delta_Baseline_FMD_t_c, residuals(meta_FMD1)) - abline(h=0) - metabias (meta_FMD1,method.bias = "linreg") - funnel (meta_FMD1) |
| --- |
| Supplement table 2. Main script for data meta-analysis in RStudio. |


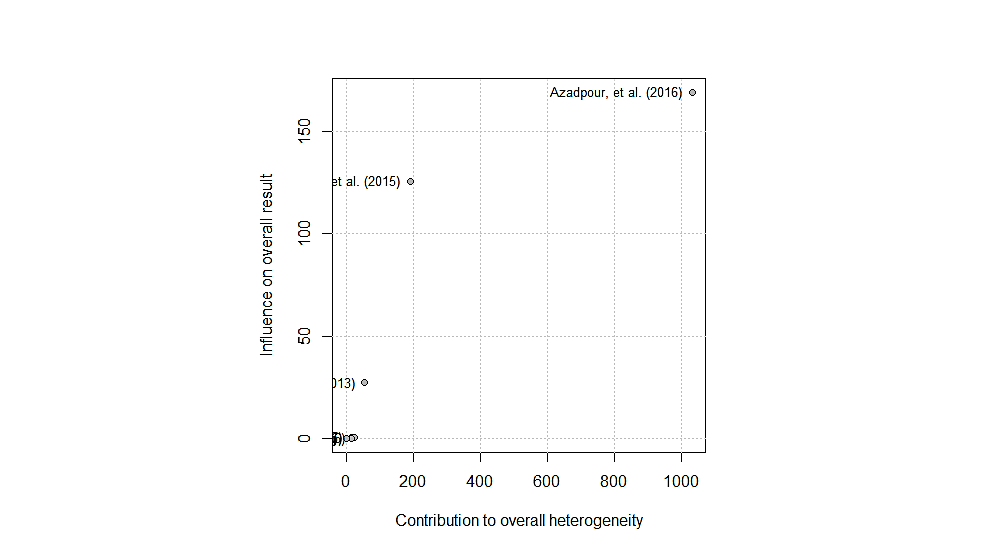


Supplement figure 1. Contribution of each study to the pooled mean result and corresponding heterogeneity of physical training in FMD in overweight individuals.


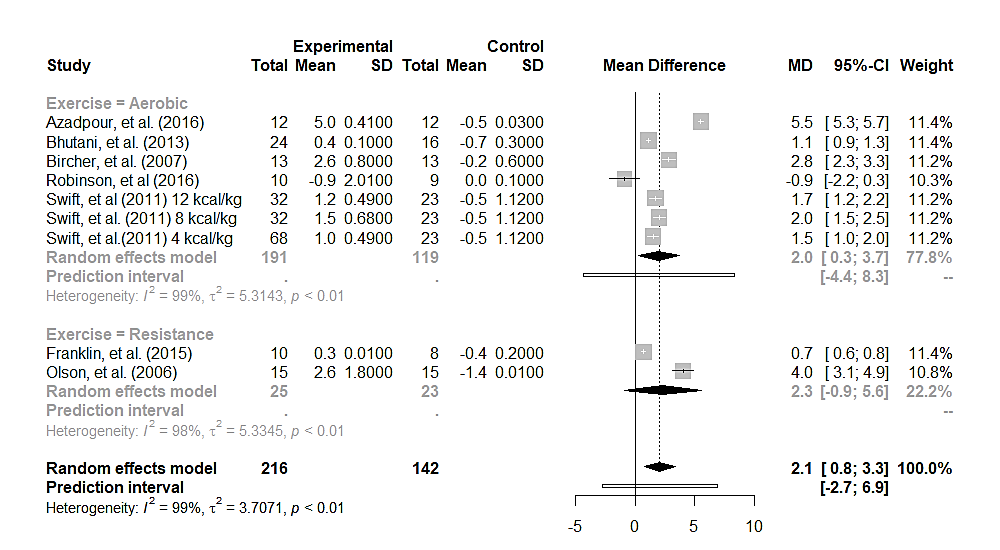


Supplement figure 2. Contribution by Azadpour et al. (2016) in FMD after physical training in overweight individuals.


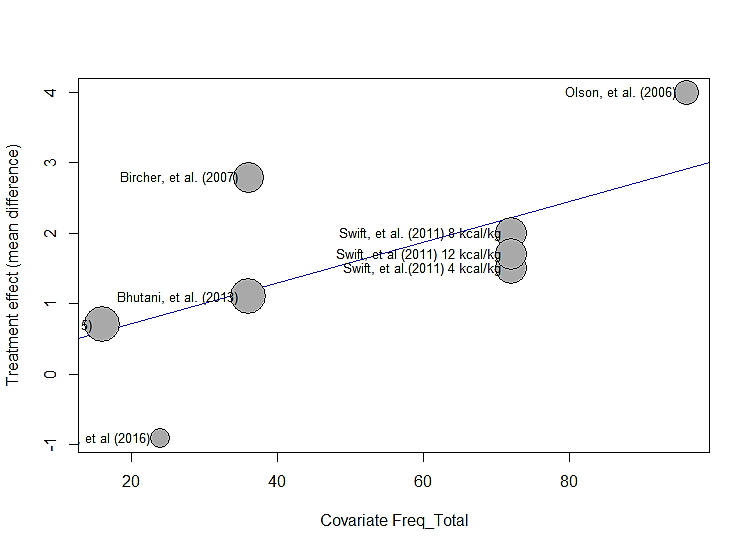

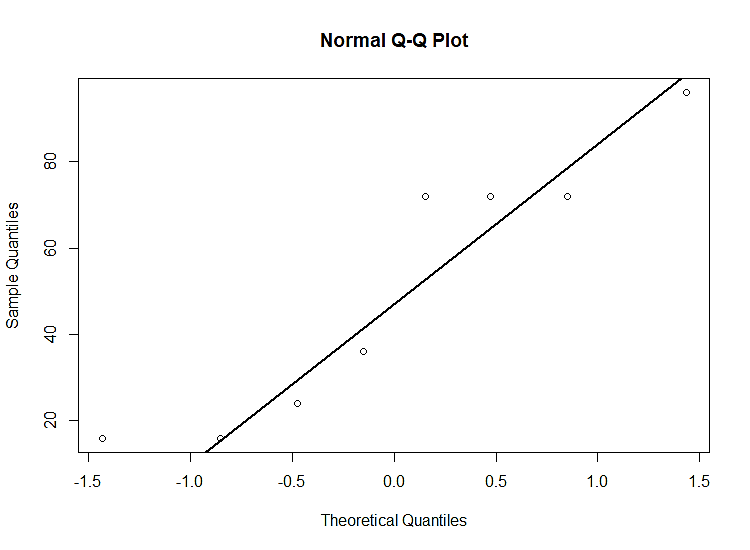


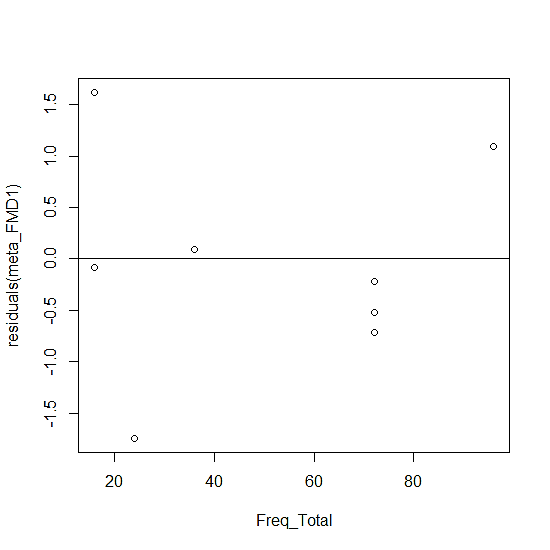


Supplement figure 3. Bubble plot of metaregression, Normal QQ Plot and Residuals Plot for “total study frequency” of overweight participants (R^2^= 44.3%, p< 0.001; hapiro Wil normality test, W= 0.868, p = 0.145).


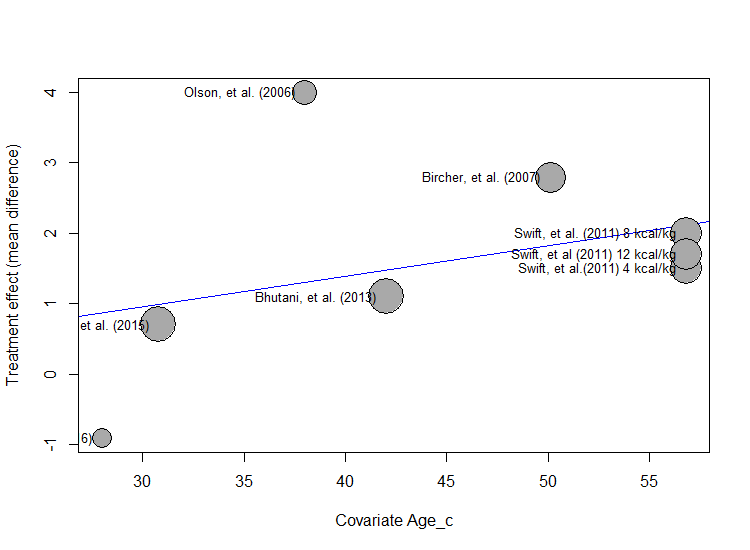


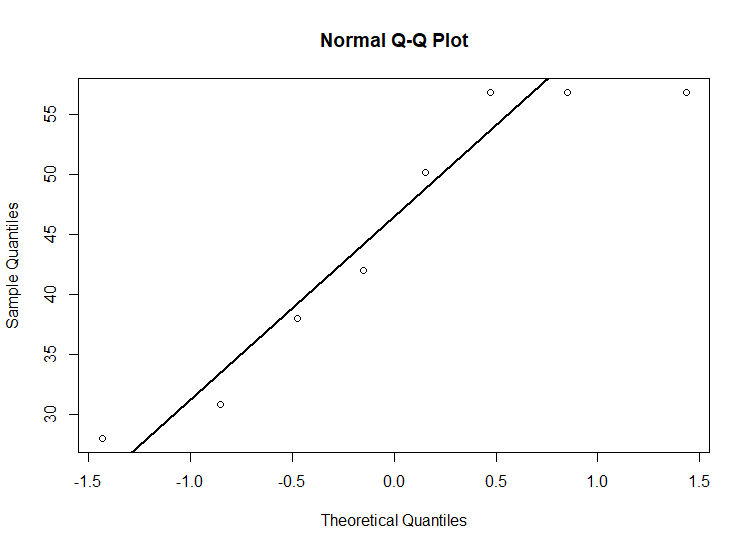


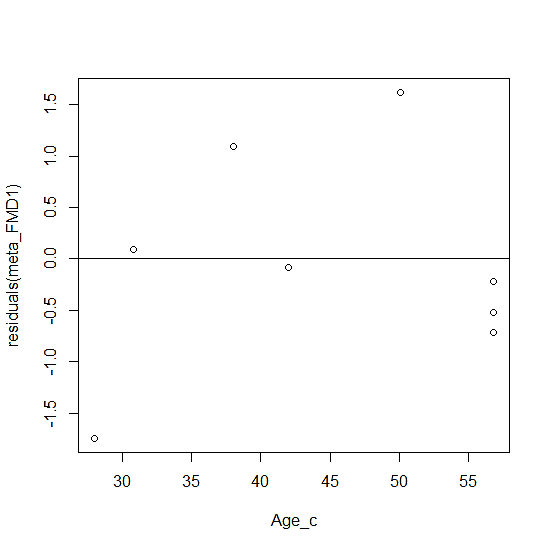


Supplement figure 4. Bubble plot of metaregression, Normal QQ Plot and Residuals Plot for “age (control group without physical training)” of overweight participants (R^2^= 24.4%, p= 0.048; Shapiro-Wilk normality test, W = 0.871, p = 0.154).


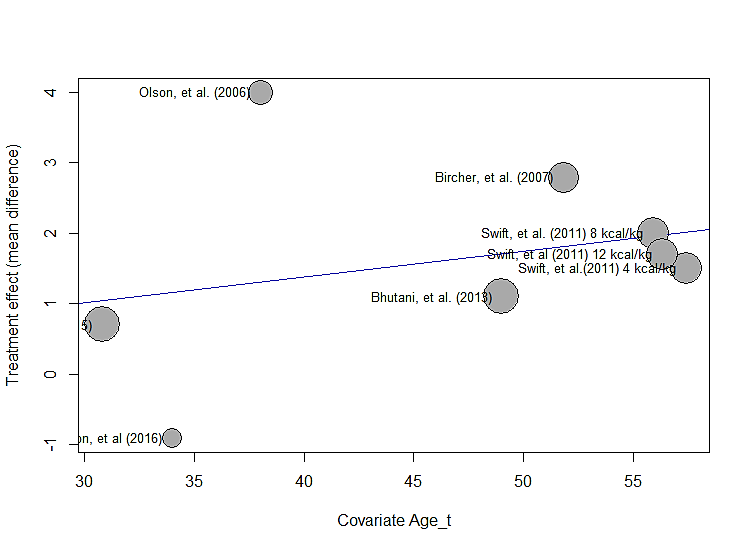


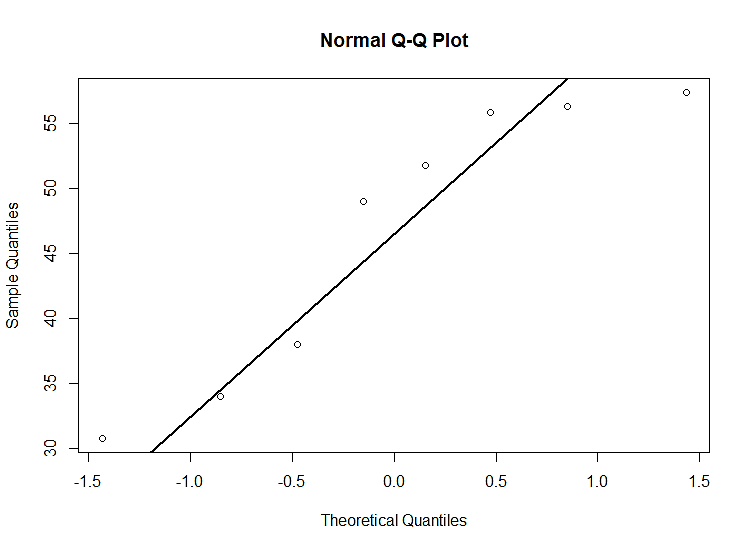


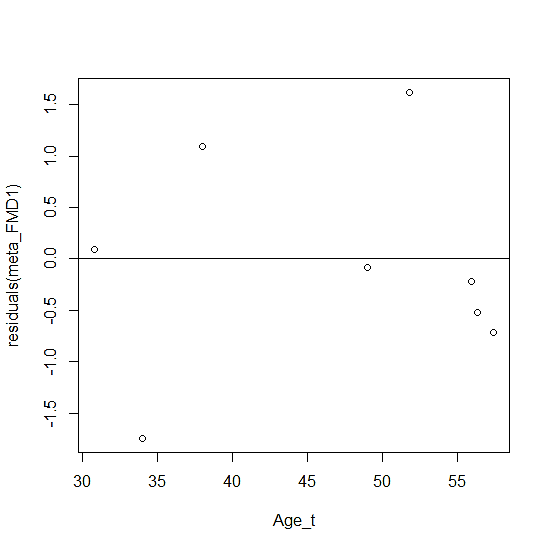


Supplement figure 5. Bubble plot of metaregression, Normal QQ Plot and Residuals Plot for “age (control group exercise training)” of overweight participants (R^2^= 00.0%, p= 0.215; Shapiro-Wilk normality test, W= 0.861, p= 0.122).


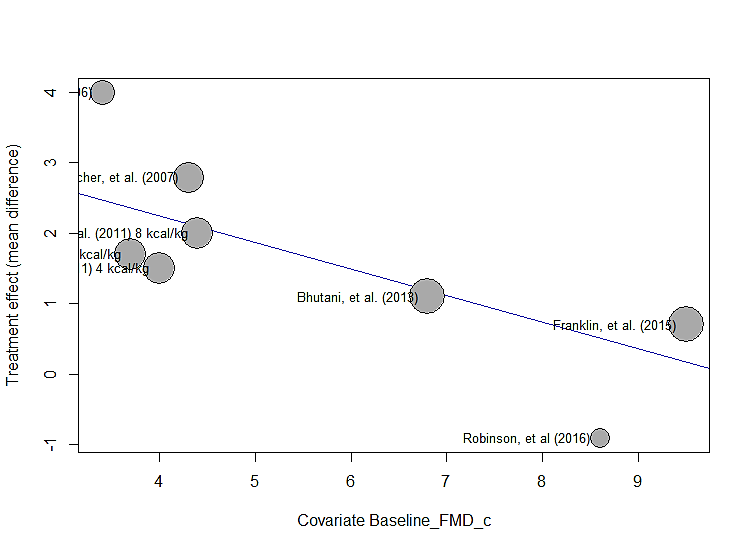


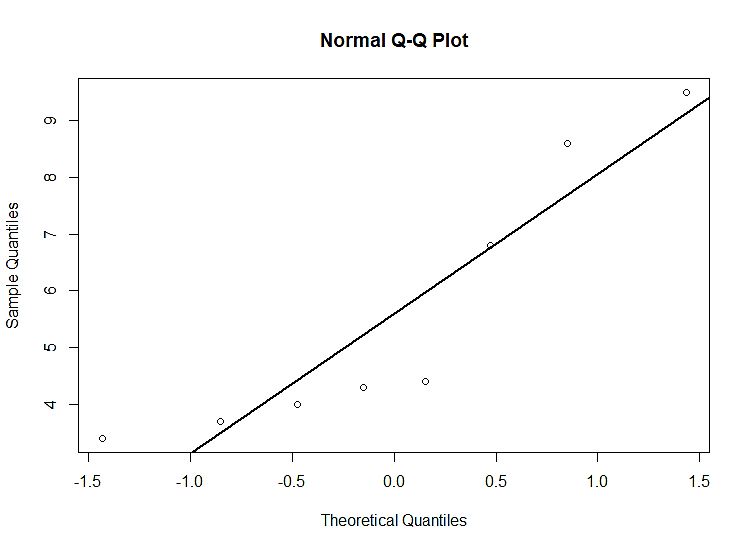


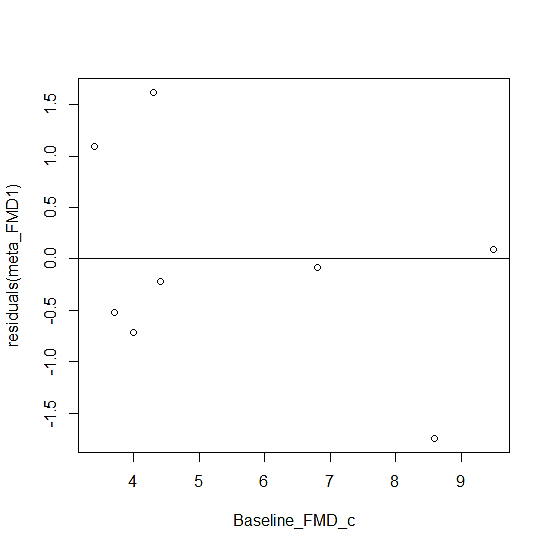


Supplement figure 6. Bubble plot of metaregression, Normal QQ Plot and Residuals Plot for “baseline FMD (control group without physical training)” of overweight participants (R^2^= 50.4%, p<0.001; Shapiro-Wilk normality test, W = 0.827, p = 0.055).


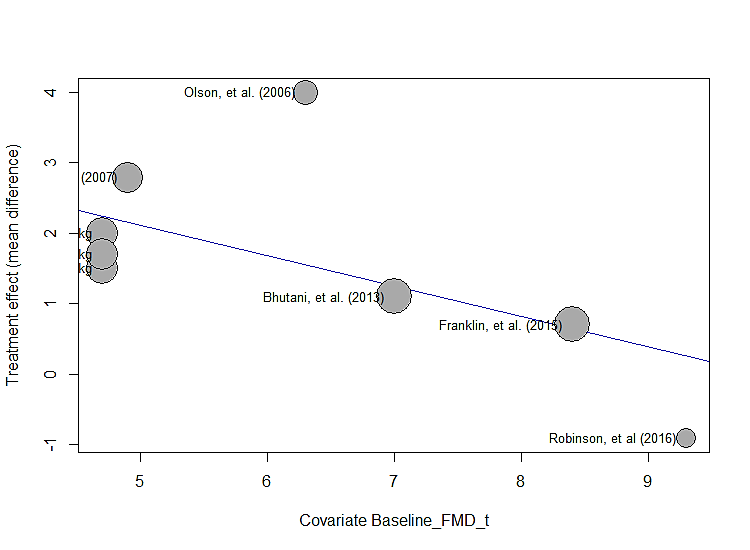


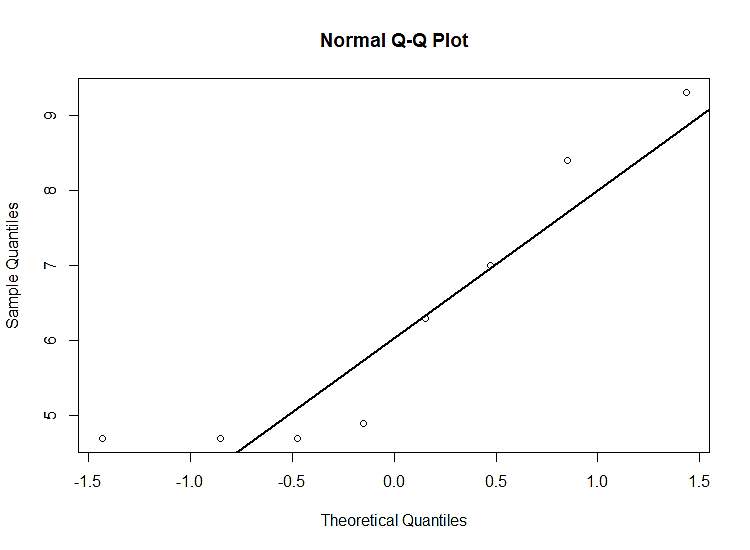


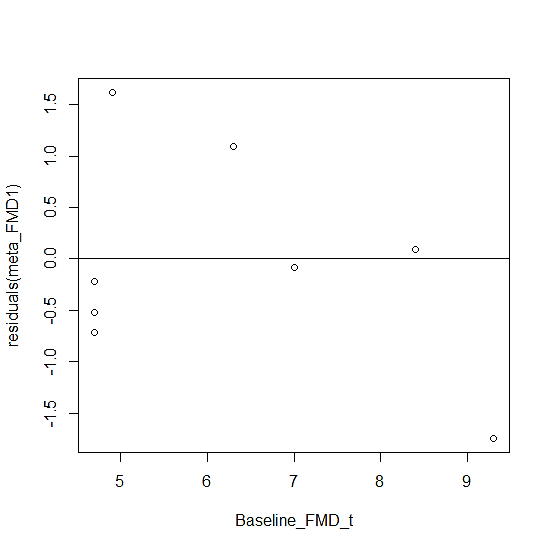


Supplement figure 7. Bubble plot of metaregression, Normal QQ Plot and Residuals Plot for “baseline FMD (physical training group)” of overweight participants (R^2^= 50.8%, p<0.001; Shapiro-Wilk normality test, W= 0.836, p=0.069).


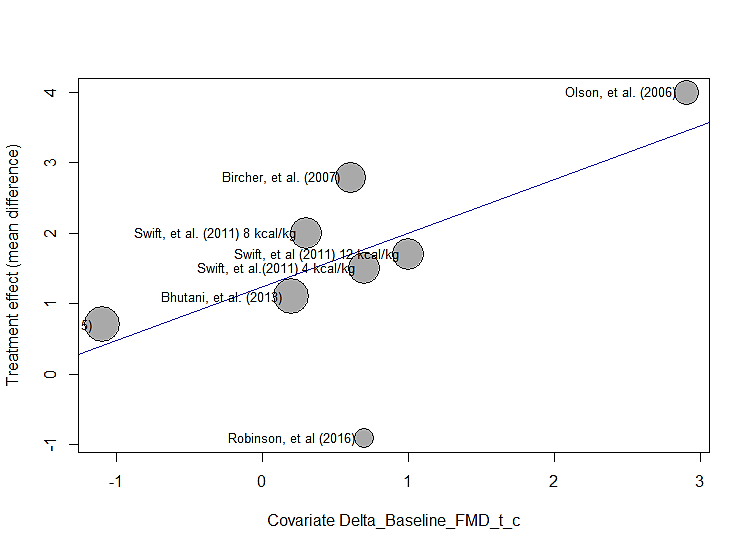

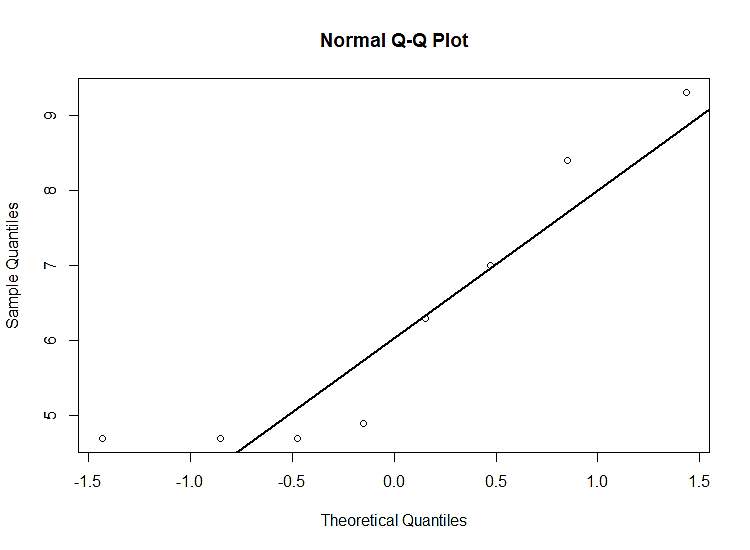


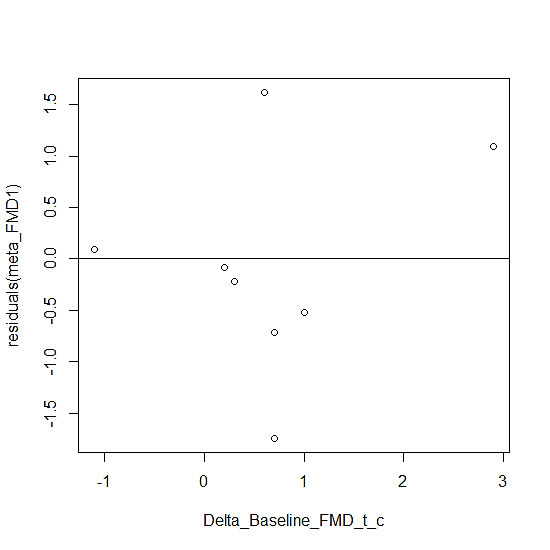


Supplement figure 8. Bubble plot of metaregression, Normal QQ Plot and Residuals Plot for “baseline FMD delta (non-trained control group vs. physical training group)” of overweight participants (R^2^= 29.3%, p<0.001; Shapiro-Wilk normality test, W= 0.881, p= 0.192)


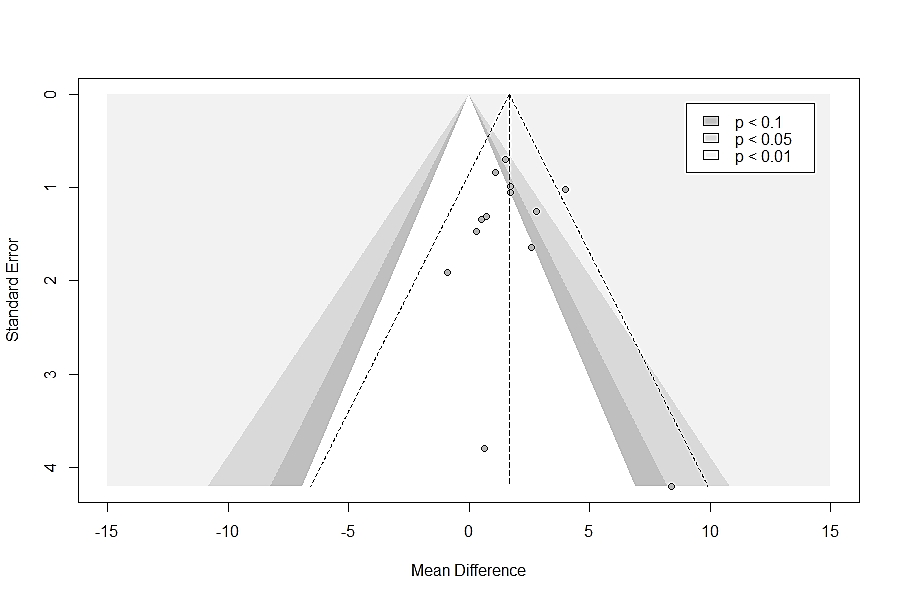


Supplement figure 9. Funnel plot and its respective p-values (t = 0.24, p-value = 0.815).
